# Chromium‐Tanned Leather and Microbial Consortia: Identification of Taxa With Biodegradation Potential and Chromium Tolerance

**DOI:** 10.1111/1758-2229.70134

**Published:** 2025-06-17

**Authors:** Manuela Bonilla‐Espadas, Irene Lifante‐Martínez, Mónica Camacho, Elena Orgilés‐Calpena, Francisca Arán‐Aís, Marcelo Bertazzo, María‐José Bonete

**Affiliations:** ^1^ INESCOP‐Footwear Technological Centre Alicante Spain; ^2^ Department of Biochemistry and Molecular Biology and Soil Science and Agricultural Chemistry, Faculty of Science University of Alicante Alicante Spain

## Abstract

Chromium‐tanned leather waste poses significant environmental challenges due to its resistance to degradation and heavy metal content. This study investigates the potential of naturally selected microbial consortia to initiate the degradation of chromium‐tanned leather and identifies key bacterial genera capable of tolerating chromium and producing enzymes relevant to collagen breakdown. A novel multidisciplinary approach combining gravimetric assays, metagenomic sequencing, and scanning electron microscopy (SEM) was applied to characterise both microbial composition and degradation dynamics. Dominant genera such as *Bacillus*, *Microbacterium*, and *Acinetobacter* were associated with collagen degradation and metal tolerance, with *Bacillus*‐rich communities showing the most pronounced mass loss (up to 3%). SEM analysis revealed the formation of robust biofilms and extensive matrix disruption, indicating enzymatic activity and structural breakdown of the leather. The formation of exopolysaccharide‐rich biofilms was found to be critical for microbial adhesion and biodegradation efficacy. These findings provide initial insights into microbial mechanisms involved in the degradation of chromium‐tanned leather and suggest potential applications for microbial consortia in future sustainable leather waste management strategies.

## Graphical abstract

- Microbial consortia naturally derived from descomposing leather initiated degradation of chromium‐tanned leather under nutrient‐limited conditions.
- Key taxa, including *Bacillus, Microbacterium* and *Acinetobacter*, formed biofilms, tolerated chromium and induced collagen matrix disruption, highlighting their potential for sustainable biotechnological applications.

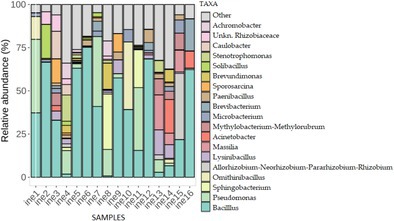

## 1 Introduction

Leather is primarily derived from raw animal hides that undergo physicochemical treatment to stabilise collagen fibres and prevent microbial degradation (Heth 2015). Collagen, the dominant protein in skin, exists in at least 28 types, with type I being the most abundant and structurally significant. This triple‐helix structure, rich in glycine, proline, and hydroxyproline, forms the basis for leather production (Shoulders and Raines 2009). Among tanning methods, chromium(III) salts are the most widely used worldwide, accounting for about 90% of global leather production due to their efficiency and ability to produce durable “wet blue” leather (Zhang, Lin, et al. 2016; Ariram et al. 2022). These salts form stable cross‐links with collagen, improving thermal and hydrolytic stability (Onem et al. 2017; Yorgancioglu et al. 2022).

Despite its industrial advantages, chromium‐tanned leather presents a significant environmental challenge (Mahmood Ali et al. 2023). During manufacturing, large volumes of solid waste are generated, including trimmings and defective products composed of chromium‐stabilised collagen (Parisi et al. 2021). These materials are highly resistant to microbial degradation and are often disposed of via landfilling or incineration, contributing to long‐term environmental contamination (Jaffari et al. 2024). The chemical complexity and toxicity of chrome‐tanned leather impede the efficiency of conventional disposal methods (Alugoju et al. 2011). Enzymatic treatments are often substrate‐specific and inefficient at the industrial scale, while chemical degradation processes can produce secondary pollutants or require harsh conditions (Khambhaty 2020; Chojnacka et al. 2021).

Alternative tanning agents such as aluminium, titanium, zirconium salts, vegetable tannins, and synthetic polymers have been proposed to address sustainability concerns (China et al. 2020). However, many of these alternatives still rely on heavy metals or produce leather with inferior mechanical properties and reduced hydrothermal stability compared to chromium‐tanned leather (Covington 2009). Moreover, these alternative leathers remain difficult to biodegrade due to persistent cross‐linked collagen networks, perpetuating challenges in solid waste management (Vico et al. 2024).

Biological approaches to managing chrome‐tanned solid waste are increasingly explored as sustainable alternatives (Akhtar et al. 2024). These include enzymatic hydrolysis, composting with adapted microbial communities, and microbial biodegradation using selected bacterial strains (Basheer and Umesh 2018; Wróbel et al. 2023). However, most studies rely on single‐species isolates under laboratory conditions, which may not replicate the complex interactions and functional diversity of natural microbial ecosystems (Verma and Sharma 2023). Furthermore, the degradation of leather is particularly challenging due to the cross‐linked collagen structure and chromium stabilisation, which hinder microbial enzymatic access and limit mass loss to below 4% after 1 month under standard testing protocols such as ISO 20136 (ISO 2020; Bonilla‐Espadas et al. 2024).

Research has increasingly focused on microorganisms capable of degrading collagenous and keratinous materials, such as *Bacillus*, *Pseudomonas* and *Acinetobacter* species (Zhang, Gong, et al. 2016; Nnolim et al. 2020). These taxa have demonstrated the production of proteases and keratinases and resistance to heavy metals, making them promising candidates for the bioconversion of leather waste (Moonnee et al. 2021; Alamnie et al. 2023; Akhtar et al. 2024). Microbial consortia, rather than pure cultures, may offer functional redundancy and synergistic effects that enhance degradation efficiency and resilience in complex waste matrices (Sheth et al. 2019).

This study aims to evaluate the biodegradation potential of naturally derived microbial consortia. Unlike previous work focusing on isolated strains or enzymatic treatments, we employed a multidisciplinary approach that combines gravimetric assays, metagenomic sequencing, and high‐resolution scanning electron microscopy (SEM). Our objectives were to (i) cultivate and select microbial consortia from decomposing leather materials, (ii) identify taxa associated with collagen degradation and metal tolerance, and (iii) assess structural and quantitative changes in leather after microbial treatment. This approach aims to expand the current understanding of microbial interactions with chrome‐tanned leather and to inform the development of sustainable, biologically based waste management strategies.

## 2 Materials and Methods

### 2.1 Inoculum Preparation and Processing

Sixteen samples from various decomposing chromium‐tanned leather pieces, as detailed in Table 1, were utilised for inoculant preparation. Inocula were obtained by placing three 1 cm × 1 cm pieces of sample in Nutrient Broth (Condalab, Madrid, Spain) for 48 h at 37°C. Once the incubation period was over, inocula were centrifuged at 4500 rpm for 5 min, washed, and resuspended in 50 mL saline solution (8.5 g NaCl/L) three times.

**TABLE 1:** Description of the 16 leather samples used for inoculant preparation, including assigned sample names.

Sample	Description
ine1	Horse leather bridle (scrapings from the top part)
ine2	Leather belt 1 (scrapings from worn part)
ine3	Leather glove 1 (scrapings from worn part)
ine4	Leather glove 1 (cut piece)
ine5	Leather reins knot
ine6	Leather belt 2 (scrapings from worn part)
ine7	Leather tool pouch
ine8	Leather glove 2 (cut piece)
ine9	Knife leather sheath
ine10	Horse leather bridle (scrapings from worn part)
ine11	Leather strip (scrapings from worn part)
ine12	Leather strip (cut piece)
ine13	Horse leather bridle strips (scrapings from worn part)
ine14	Horse leather bridle strips (cut piece)
ine15	Leather belt 1 (cut piece)
ine16	Leather strip (scrapings from darker part)

### 2.2 Inoculated Flask Setup and Incubation

Individual experiments were prepared in 150 mL flasks using 16 inoculants (ine1 to ine16) obtained in 2.1 each tested in triplicate (*n* = 48 experimental flasks) as shown in Table 2. For each replicate, three 1 cm × 1 cm cut pieces of chromed semifinished leather (wet blue) (Biškauskaitė and Valeika 2023) were placed in 50 mL for each experiment. The M9 minimal medium (ATCC 2511) (Merck Life Science S.L.U., Madrid, Spain) was prepared according to the manufacturer's instructions. M9 salt medium and leather samples were placed as a negative control to rule out possible growth from carbon sources in the inoculum. The leather pieces were weighed using a precision balance before incubation to allow calculation of percentage weight loss. All flasks were incubated statically at 30°C for 8 weeks.

**TABLE 2:** Qualitative microbial growth in flasks over time in samples ine1 to ine16 (1, 2 and 3 represent the three replicates of all samples).

Sample	Week 2			Week 5			Week 8		
	1	2	3	1	2	3	1	2	3
ine1	++	++	++	+++	+++	+++	+++	+++	+++
ine2	+	+	++	++	++	+++	++	++	+++
ine3	+	+	+	+	+	+	+	++	++
ine4	+	++	++	+++	+++	+++	+++	+++	+++
ine5	+	+	+	+	+	+	+	++	++
ine6	+	+	+	+	+	+	+	+	+
ine7	−	−	+	+	+	+	+	++	++
ine8	−	−	−	−	−	−	−	−	−
ine9	+	+	+	++	++	++	++	++	++
ine10	−	−	−	+	+	+	+	+	+
ine11	+	+	+	++	++	++	++	++	++
ine12	++	++	+++	+++	+++	++++	+++	+++	+++
ine13	+	++	+	++	+++	++	++	+++	++
ine14	++	++	++	+++	+++	+++	+++	+++	+++
ine15	+	++	+	+++	+++	++	+++	+++	+++
ine16	++	++	++	++	++	++	++	++	++

*Note:* Key: “−” no growth, “+” low turbidity, “++” moderate turbidity, “+++” high turbidity, “++++” very high turbidity. Growth was assessed at weeks 2, 5, and 8 based on medium turbidity.

### 2.3 Optical Density Qualitative Monitoring

An optical density qualitative monitoring of the medium was conducted throughout the incubation period, assigning “−” to no growth or “+,” “++,” “+++,” or “++++” to growth based on the turbidity present in the flasks compared to the negative control (Table 2). After the incubation period, no further monitoring was conducted on replicate 1 of each of the 16 samples since they were used for DNA extractions.

### 2.4 Metagenomic DNA Extraction From Inoculated Flask Cultures

Genomic DNA extraction from each sample was performed with NZY Soil gDNA Isolation Kit (NZYtech, Lisboa, Portugal) following the manufacturer's instructions. DNA was extracted from replicate 1 of each flask after 8 weeks of incubation, which was sacrificed for this purpose and therefore excluded from subsequent analysis. The three leather pieces were taken using flame‐sterilised tweezers and placed in Falcon‐type tubes with 1 mL of Phosphate Buffer Saline (PBS). The mixture was vigorously vortexed for approximately 2–3 min; the liquid was transferred to an Eppendorf‐type tube and centrifuged for 3 min at 12200 rpm. The supernatant was discarded, and the pellet was collected and transferred to a bead tube. A small piece of the leather was cut with a scalpel and placed in the bead tube. According to the standard protocol, DNA was quantified through a Qubit dsDNA Quantification Assay kit (ThermoFisher, Waltham, Massachusetts, United States).

### 2.5 Metagenomic Identification and Bioinformatics Analysis

The conserved V3 and V4 regions (459 bp) of the 16S rRNA gene were amplified using the forward primer 5′‐TCG TCG GCA GCG TCA GAT GTG TAT AAG AGA CAG CCT ACG GGN GGC WGC AG‐3′ and the reverse primer 5′‐GTC TCG TGG GCT CGG AGA TGT GTA TAA GAG ACA GGA CTA CHV GGG TAT CTA ATC C‐3′. Amplification was performed with the KAPA HiFi HotStart ReadyMix (Roche, Basel, Switzerland) under the following conditions: initial denaturation at 95°C for 3 min; 25 cycles of 30 s at 95°C, 30 s at 55°C and 30 s at 72°C; and a final extension at 72°C for 5 min. Amplicons were combined with Illumina sequencing adaptors and dual‐index barcodes using the Nextera XT Index Kit v2 (Illumina, San Diego, California, United States). After normalisation and merging, the indexed amplicons were loaded onto the MiSeq Reagent Kit v3 (Illumina, San Diego, California, United States) and spiked with 10% PhiX control to improve sequencing quality. Paired‐end sequencing (2 × 300 bp) was conducted on the Illumina MiSeq System at the Foundation for the Advancement of Health and Biomedical Research of the Valencian Community (Fisabio) in Valencia, Spain.

The raw sequences generated by Illumina were imported into the bioinformatics tool Qiime2 (Bolyen et al. 2019) for an initial sequence quality control process using DADA2. Taxonomic assignment of each amplicon sequence variant (ASV), defined at a 99.9% sequence similarity, was performed using the classify‐Sklearn module in combination with the SILVA v138 database (Quast et al. 2013). Statistical and microbial ecology analyses were conducted using various R software packages; rarefaction curves were generated using the iNEXT and ggiNEXT functions from the iNEXT package (Hsieh et al. 2016). Alpha diversity plots, including richness (Walters and Martiny 2020), Shannon (Feranchuk et al. 2018), and Simpson (Kim et al. 2017) diversity indexes, were created with the plot richness function in Phyloseq (McMurdie and Holmes 2013) and Vegan (Oksanen et al. 2022). The Wilcoxon Rank Sum Test was used to assess significant differences between sample groups at the alpha diversity level. The DESeq2 test was employed (Love et al. 2014) to determine significant differences in the relative abundances of taxa. Beta diversity analysis was conducted using principal component analysis (PCoA) after calculating a dissimilarity matrix between samples with the Bray‐Curtis method (Beals 1984). PERMANOVA statistic (Anderson 2017) was used to detect statistically significant differences in microbiome composition among the analysed groups.

### 2.6 Identification of Cultivable Bacterial Strains

In parallel to the metagenomic identification of bacterial strains, the most promising flasks based on criteria such as flask turbidity and cell abundance seen under the microscope, samples ine12, ine14 and ine15 (replicates 2 and 3 of each sample) were selected to be cultured so that cultivable strains present could be identified. These samples were plated on Tryptic Soy Broth (TSB) media with 3% and 1.5% agar, as well as on R2A medium (0.1% peptone; 0.05% yeast extract; 0.05% glucose; 0.05% starch; 0.03% K_2_HPO_4_; 0.005% Mg_2_SO_4_·7H_2_O; 0.03% sodium pyruvate). Bacterial identification was carried out through partial sequencing of the 16S rRNA gene following the methodology outlined in Bonilla‐Espadas et al. (2024).

### 2.7 Gravimetric Analysis

After an 8‐week incubation period, the three leather pieces placed in each of the 16 flasks (replicate 1 of each sample) were weighed to determine the percentage of mass loss. The leather pieces were washed in a 10% SDS solution with agitation for 24 h, then rinsed with sterile distilled water for 2 h to remove bacteria and any biofilms formed on the surface. Following this, the samples were incubated in 70% ethanol with agitation for another 2 h and then dried for 72 h. Finally, the leather pieces were weighed, and the percentage of mass loss was calculated to the negative control.

### 2.8 Characterisation of Biofilm Formation Using SEM

Images were taken using the high‐resolution technique SEM following the methodology outlined by Vyskočilová et al. (2022) for samples ine12, ine14 and ine15, and negative controls were used to conduct a comparative analysis of leather surface morphology, leather degradation, and the presence and absence of biofilm formation.

## 3 Results

### 3.1 Refraction

**FIGURE 1:**
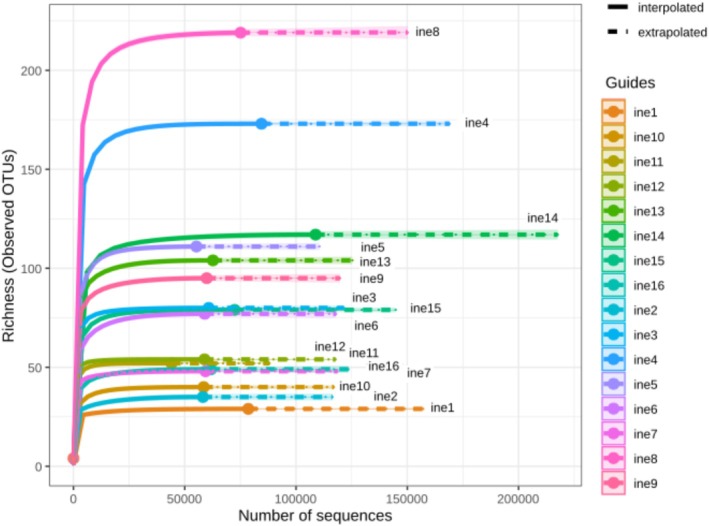
Rarefaction curves showing observed richness (number of ASVs) as a function of sequencing depth for all samples (ine1 to ine16). Curves were generated at the amplicon sequence variant (ASV) level. The plateau in most curves indicates sufficient sequencing coverage for capturing microbial diversity within each inoculant‐derived flask culture.

To assess the microbial diversity in the inoculated cultures, we first examined species richness, which refers to the number of distinct amplicon sequence variants (ASVs) identified per sample. Rarefaction curves (Figure 1) show that all samples reached a plateau, indicating sufficient sequencing depth to capture most microbial diversity in each case.

### 3.2 Alpha and Beta Diversity

**FIGURE 2:**
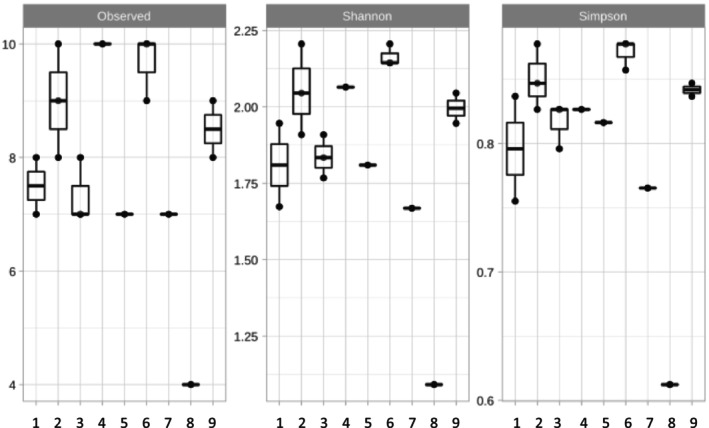
α‐diversity at the ASV (Amplicon Sequence Variant) level grouped by sample type. Samples; 1: Horse leather bridle (ine1, ine10), 2: Leather strip (ine11, ine12, ine16), 3: Leather belt (ine2, ine6, ine15), 4: Knife leather sheath (ine9), 5: Leather tool pouch (ine7), 6: Leather glove (ine3, ine4, ine8), 7: Leather reins knot (ine5), 8: SeqControl (Chong et al. 2014) control used in sequencing, 9: Horse leather bridle strips (ine13, ine14).

Diversity analysis at the ASV level, grouped by sample type, revealed variation in richness, Shannon, and Simpson diversity indices (Figure 2). A Wilcoxon Rank Sum Test found no significant differences in richness, Simpson index, or Shannon index between groups (*p* > 0.05 in all cases). Due to sample grouping variability, some categories (e.g., tool pouch, knife sheath) contain only one representative sample.

**FIGURE 3:**
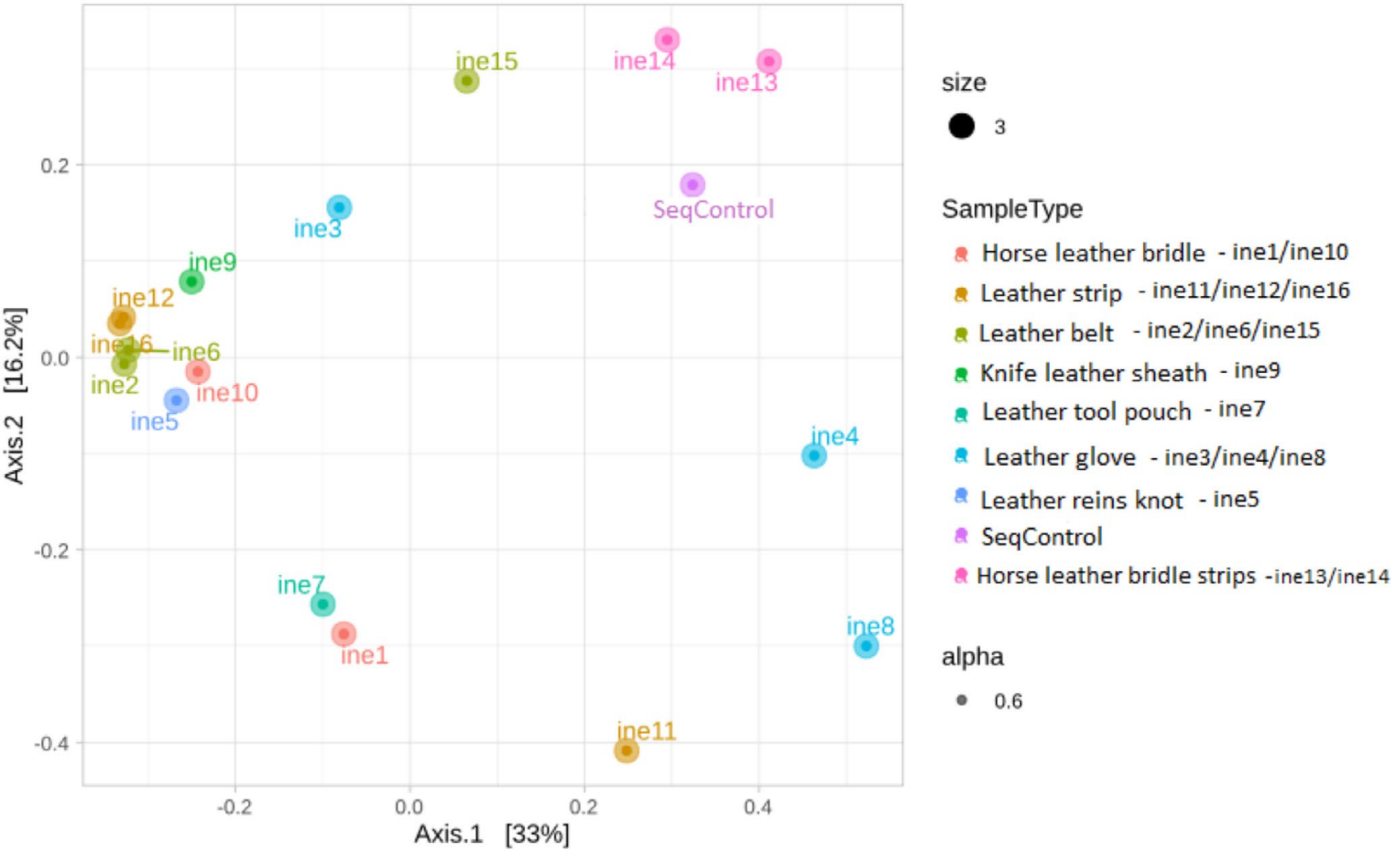
β‐diversity in all samples (ine1 to ine16). SeqControl (Chong et al. 2014) control is used in sequencing.

At the β‐diversity level shown in Figure 3, certain sample groups, such as leather strips (ine12, ine16), belts (ine2, ine6), and gloves (ine3, ine4), exhibit clustering patterns where two samples cluster closely, indicating a similar microbial composition, while a third sample (ine11, ine15 and ine8, respectively) is more distantly related. In contrast, the two samples from bridle strips cluster tightly together, whereas the broader bridle samples show more dispersion. Additionally, samples ine12 and ine16 (leather strips), ine2 and ine6 (belts), ine9 (knife sheath), ine10 (bridle), and ine5 (rein knot) demonstrate a very similar microbiome, as indicated by their proximity on the PCoA plot. A PERMANOVA test reveals significant microbiome differences across the different sample types (*p*‐value < 0.05), highlighting distinct microbial compositions among these groups.

### 3.3 Determination of Microbial Taxa

**FIGURE 4:**
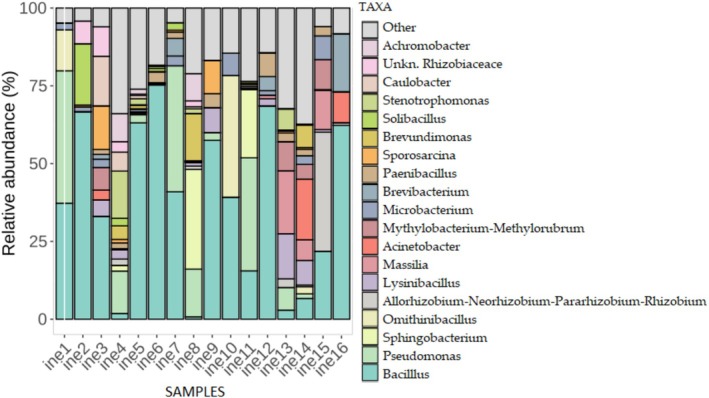
Taxonomic composition of bacterial communities at the genus level in samples ine1 to ine16.

Figure 4 illustrates the relative abundance of each bacterial genus for all samples (ine1 to ine16). *Bacillus* emerged as the predominant genus present in all samples, with exceptionally high abundance in ine6 and ine12, while only trace amounts were detected in samples like ine4 and ine8. *Pseudomonas* was prevalent in ine1, ine7, ine8 and ine11, while *Acinetobacter* showed high abundance in ine14 and ine16. Other notable genera included *Ornithinibacillus*, particularly in samples ine1, ine10 and ine14.

### 3.4 Identification of Cultivable Strains

Alongside the metagenomic analysis, samples ine12, ine14 and ine15 were selected to isolate the most abundant bacterial strains. These samples were chosen based on their growth density, taxonomic diversity, and the origin of the inoculum. The bacterial strains identified in these consortia are detailed in Table 3.

**TABLE 3:** Cultivable bacterial strains isolated from samples ine12, ine14 and ine15.

Sample	Medium	Closest species	Similarity
ine12	TSB	*Microbacterium paraoxydans* strain CF36 (NR_025548.1)	99.1%
ine12	TSB	*Rhodococcus soli* strain DSD51W (NR_134799.2)	99.6%
ine14	TSB	*Lysinibacillus fusiformis* strain DSM 2898 (NR_042072.1)	99.5%
ine14	TSB	*Aeromicrobium tamlense* strain SSW1‐57 (NR_043791.1)	99.7%
ine14	TSB	*Microbacterium lacticum* strain DSM 20427 (NR_026160.1)	98.8%
ine15	TSB	*Brevibacterium casei* strain DSM 20657 (NR_041996.1)	98.8%
ine15/ine14	TSB	*Microbacterium maritypicum* strain DSM 12512 (NR_114986.1)	99%
ine12	R2A	*Cellulomonas pakistanensis* strain NCCP‐11 (NR_125452.1)	99.7%
ine12	R2A	*Bacillus pumilus* strain NRRL NRS‐272 (NR_116191.1)	99.3%
ine14	R2A	*Pigmentiphaga litoralis* strain JSM 061001 (NR_044530.1)	98.3%
ine14	R2A	*Microbacterium aurum* strain DSM 8600 (NR_044933.1)	99.2%

*Note:* The table lists the origin sample, the culture medium used, the closest species identified via 16S rRNA gene sequencing, the corresponding NCBI RefSeq accession number, and the percentage of sequence similarity.

The predominant genus among the cultivable samples is *Microbacterium*. Other genera, such as *Bacillus* and *Lysinibacillus*, have also been successfully cultivated.

### 3.5 leather Sample Mass Loss

**FIGURE 5:**
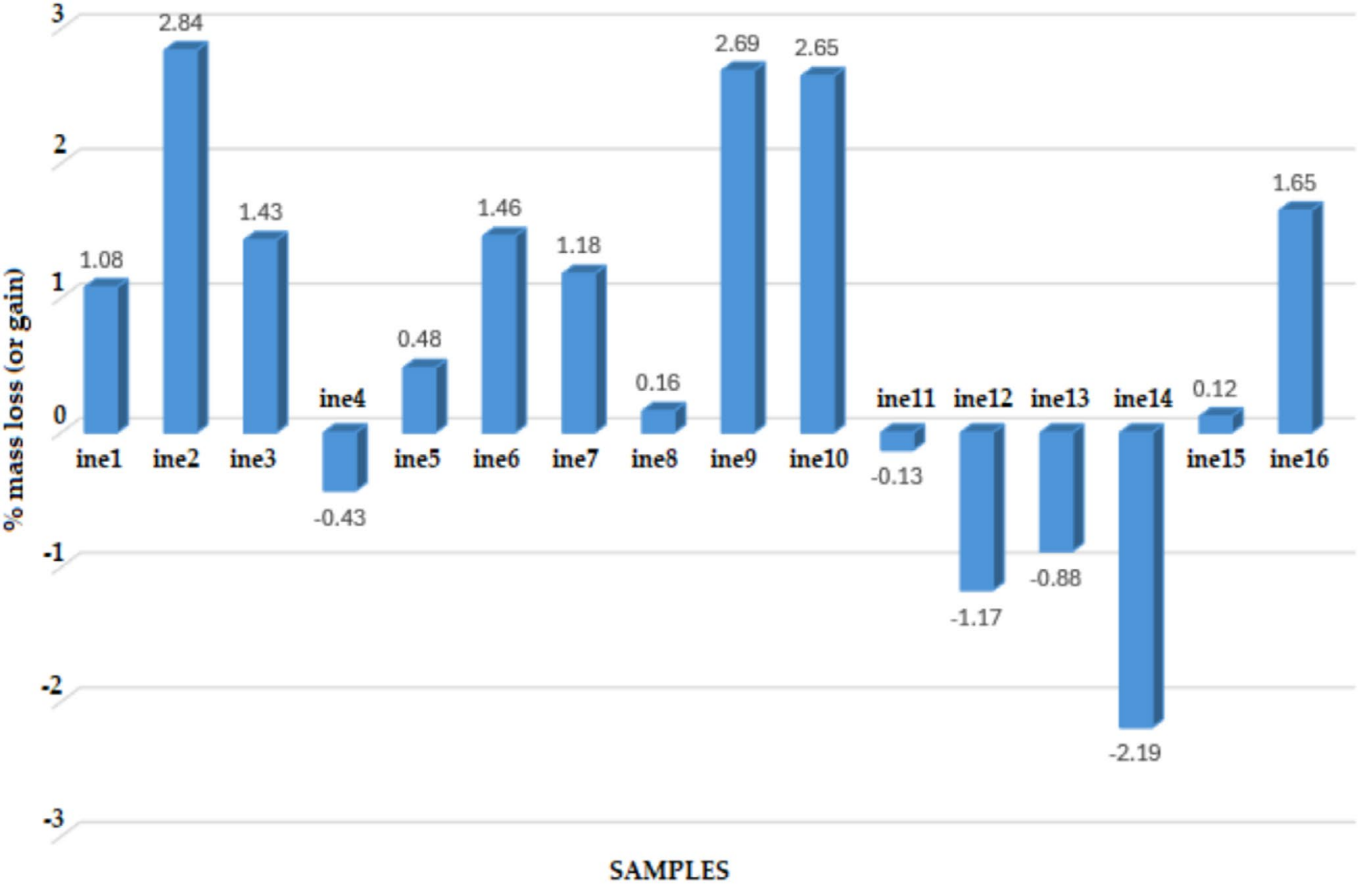
Percentage of mass loss (or gain) for each leather sample (ine1–ine16) after microbial treatment relative to the corresponding negative control. Positive values indicate material degradation, whereas negative values may reflect variability in surface biomass removal or sample hydration following the washing and drying protocol.

As shown in Figure 5, approximately half of the microbial consortia (ine1 to ine16) exhibited mass losses ranging from 1% to 3%, with samples ine2, ine9, and ine10 demonstrating the highest levels of degradation. These samples also displayed elevated relative abundances of *Bacillus*, *Microbacterium*, and *Ornithinibacillus* (Figure 4). Similarly, samples ine3, ine6, and ine16, which also showed a notable mass loss, show a high presence of *Bacillus* and *Acinetobacter*, previously recognised as effective leather degraders. In contrast, specific samples (ine4, ine11, ine12, ine13, and ine14) displayed negative mass loss percentages. Importantly, no external carbon source was provided beyond leather itself.

### 3.6 Characterisation of Biofilm Formation Using SEM

**FIGURE 6:**
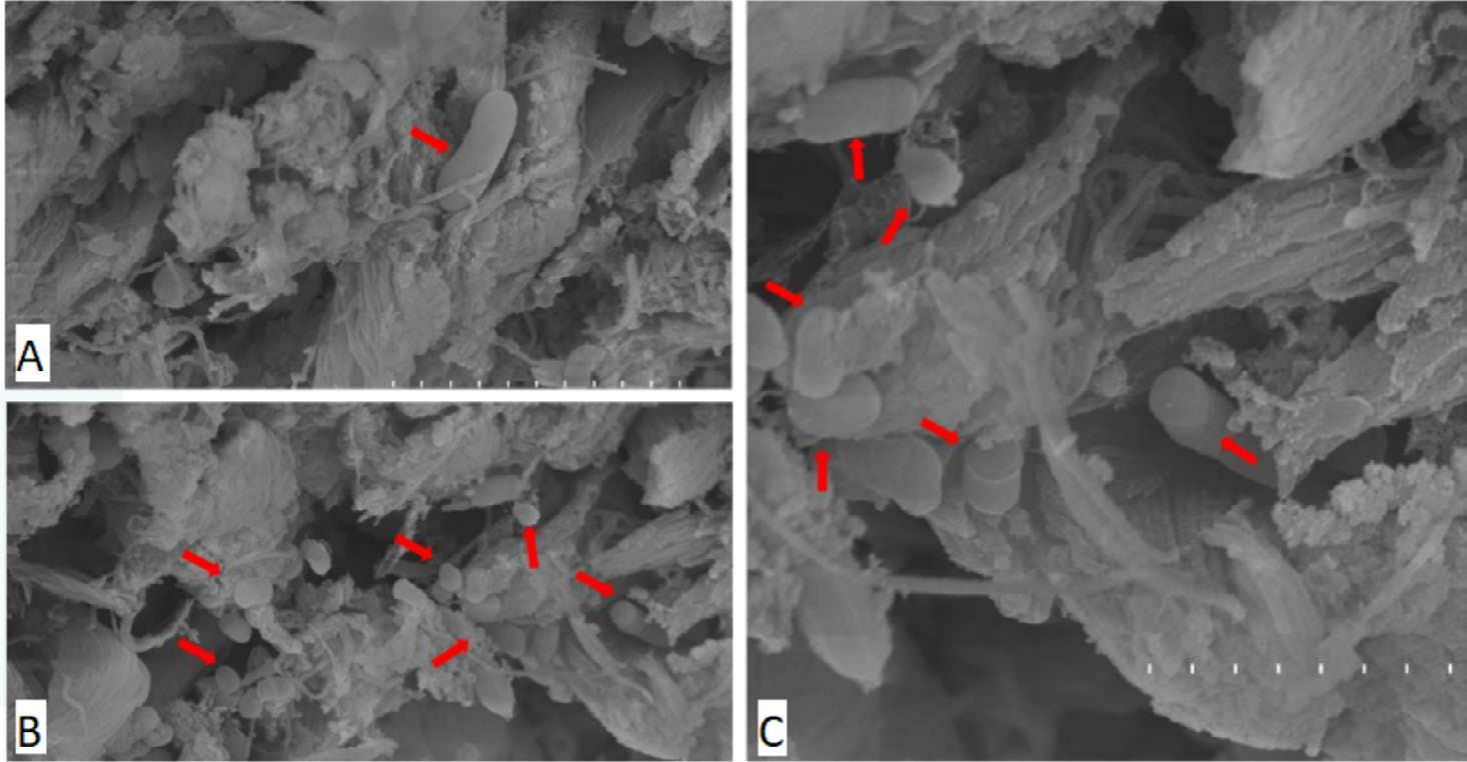
Scanning electron microscope (SEM) images of sample ine12 highlighting adhered bacteria to the leather sample. Red arrows indicate bacterial adhesion sites, with no substantial evidence of biofilm formation or exopolysaccharide (EPS) production. (A) Magnification ×15000 showing scattered bacterial adhesion. (B) Magnification ×5000 provides a broader view of bacteria interacting with the leather substrate. (C) Magnification ×25000 offering detailed visualisation of bacterial attachment.

**FIGURE 7:**
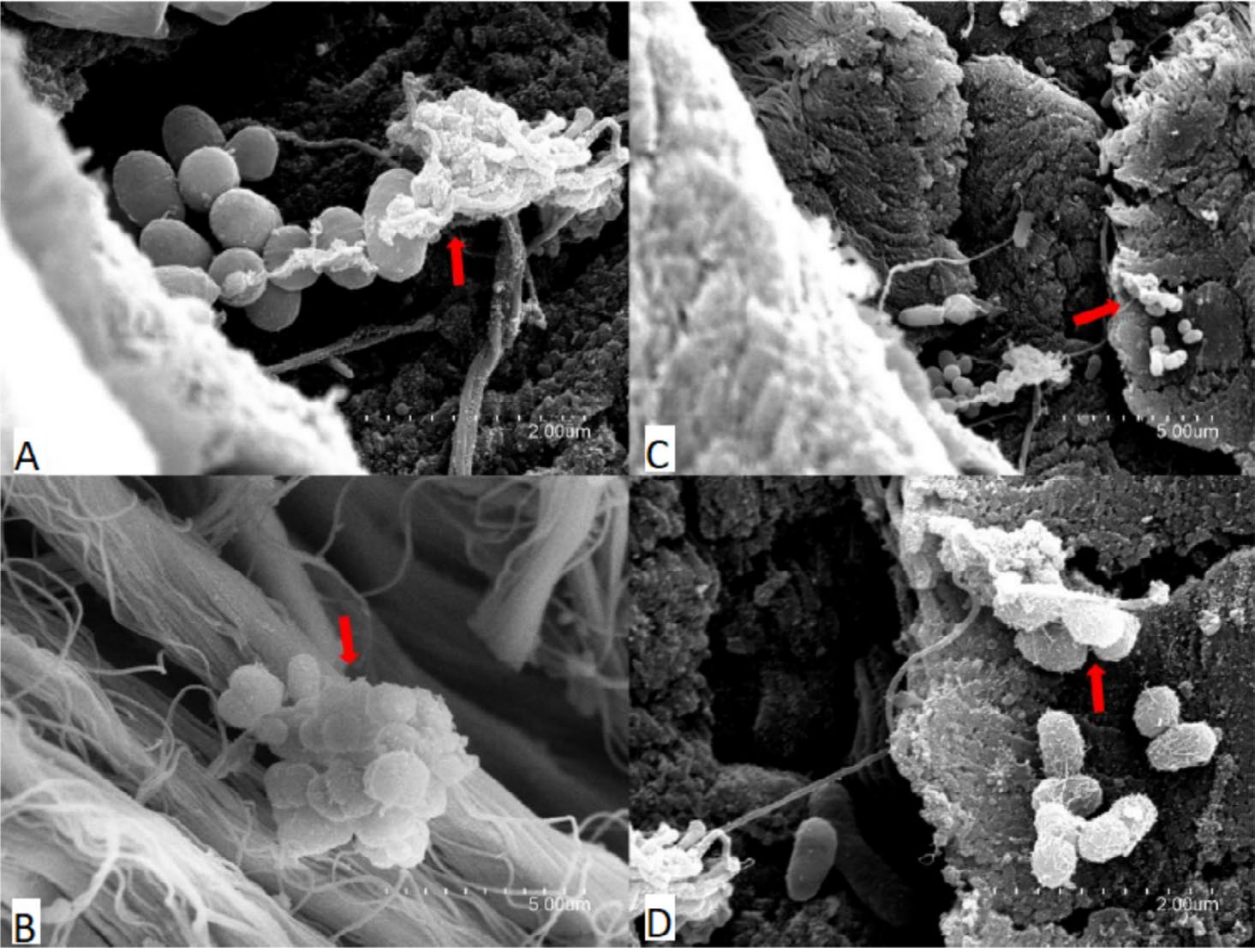
Scanning electron microscope (SEM) images of sample ine14 highlighting biofilm structures and exopolysaccharide (EPS) production. Red arrows indicate areas of biofilm localization and EPS presence. (A) Magnification ×15000 shows cohesive biofilm structures. (B) Magnification ×25000 shows clear evidence of EPS production contributing to biofilm integrity. (C) Magnification ×5000 focuses on EPS interaction with the leather substrate. (D) Magnification ×15000 provides a detailed visualisation of biofilm morphology and material interaction.

**FIGURE 8:**
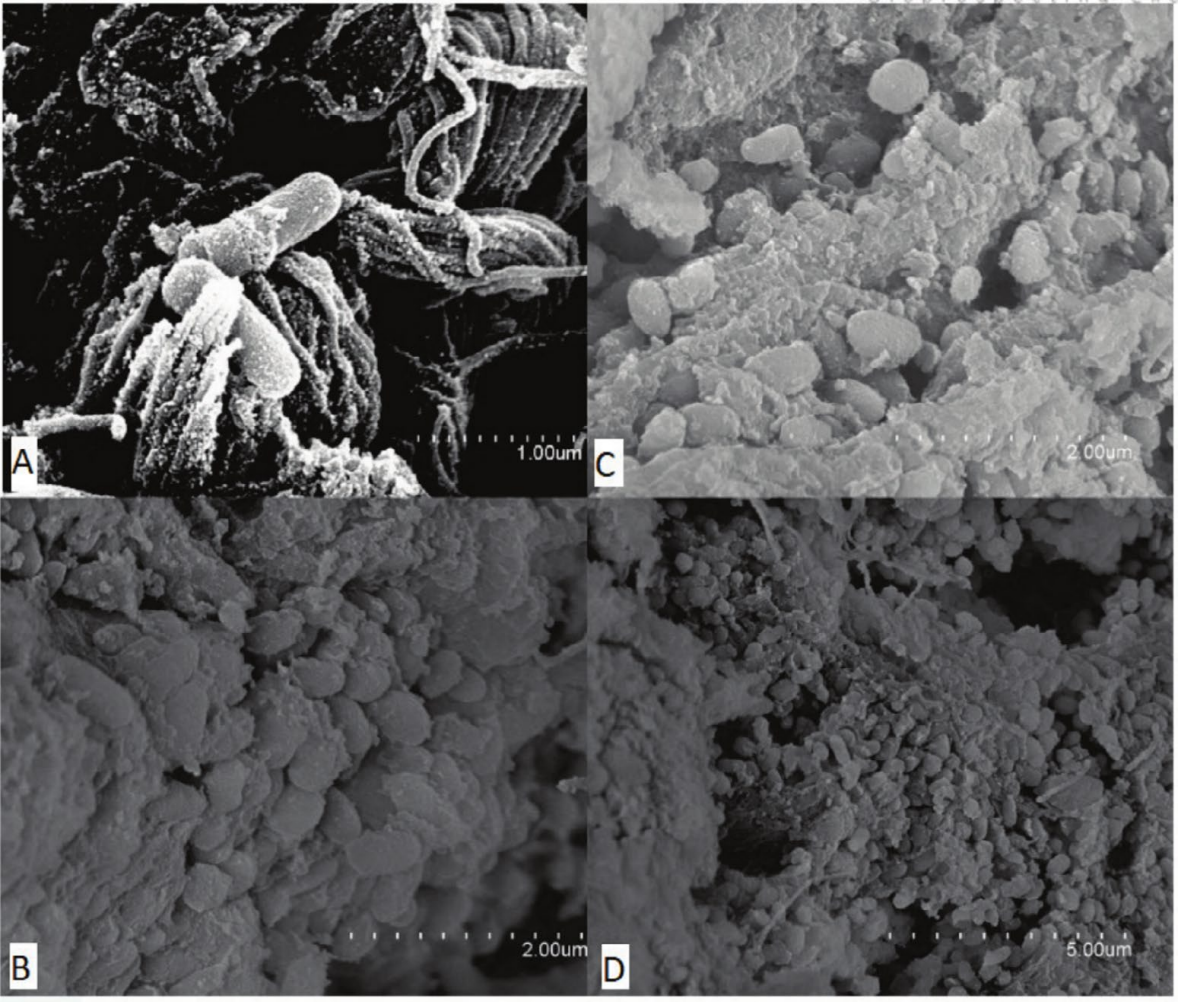
Scanning electron microscope (SEM) images of sample ine15 illustrating microbial activity and material interactions. (A) Magnification ×25000, highlighting bacterial adhesion to leather fibres. (B) Magnification ×15000 showing clusters of adhered microorganisms. (C) Magnification ×10000 provides a broader view of microbial colonisation. (D) Magnification ×5000 reveals the distribution of microbial communities across the sample surface.

**FIGURE 9:**
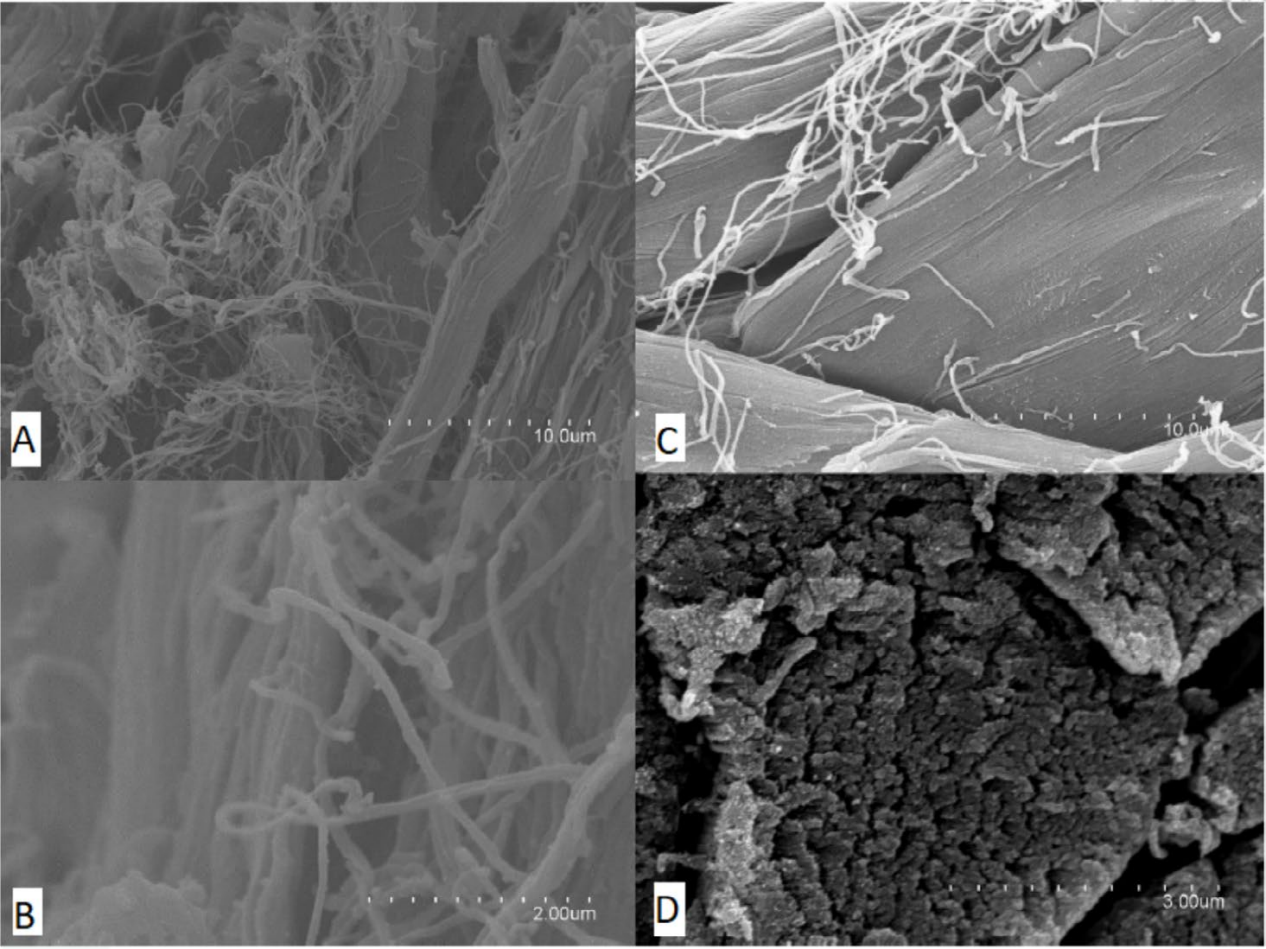
Scanning electron microscope (SEM) images of the negative control leather sample highlighting the structural integrity and typical periodic arrangement of collagen fibrils. (A) Magnification ×15000 shows the periodic structure of collagen fibrils. (B) Magnification ×50000 provides a closer view of the intact fibrillar network. (C) Magnification ×25000 shows a well‐ordered fibrillar collagen network. (D) Magnification ×10000 highlighting the absence of microbial activity or surface deformation.

SEM was employed to assess surface degradation and microbial colonisation of leather samples, providing high‐resolution visual evidence of biofilm formation and matrix disruption during microbial activity. The leather samples selected for SEM analysis (ine12, ine14 and ine15) were previously designated as cultivable inoculations, as detailed in Section 2.6. These samples were initially identified as the most promising due to their high turbidity and visible microbial abundance under the microscope, making them appropriate for strain isolation and structural examination. Although not chosen based on gravimetric performance, they reflect distinct biodegradation stages: ine12 and ine14 exhibited negligible or negative mass loss, whereas ine15 showed more advanced structural disruption. SEM imaging (Figures 6, 7, 8) revealed varying degrees of collagen matrix deterioration, including discontinuous and loosened fibre cohesion, progressive shrinkage, and extensive melted or gelatinized areas. These morphological changes are consistent with the loss of periodic ultrastructural arrangement of collagen fibrils, as Bozec and Odlyha (2011) described. Fibrillar structures appeared amorphous and gelatinised at higher magnifications (e.g., Figures 6C and 8C), further supporting microbial activity. In contrast, the negative control sample (Figure 9) preserved the typical periodic arrangement and structural integrity of collagen fibrils, confirming the absence of microbial activity and serving as a baseline for comparison. While this selection enabled the visualisation of microbial colonisation across different degradation levels, future analyses should include additional samples, such as ine2, ine8, and ine10, to more robustly assess the relationship between biofilm formation and leather mass loss.

## 4 Discussion

This study provides a multidisciplinary assessment of microbial consortia derived from environmental leather samples and their role in initiating chromium‐tanned leather degradation. The integration of metagenomics, strain cultivation, gravimetric analysis, and SEM has enabled a comprehensive understanding of the microbial activity, diversity, and associated structural effects on leather. Rarefaction and alpha diversity analyses confirmed the richness and evenness of the microbial communities within the flask cultures. All samples reached a sequencing plateau, indicating a sufficient depth to capture the majority of ASVs (Walters and Martiny 2020). Samples ine8, ine4 and ine14 displayed higher richness, suggesting more complex or diverse inoculant communities, possibly influenced by the nature or exposure history of the leather samples. Conversely, samples ine1 and ine2 showed lower richness, reflecting less complex microbial profiles.

Beta diversity patterns revealed distinct clustering based on the leather type and usage history. The PERMANOVA results confirmed that the microbial community composition varied significantly between sample groups (Anderson 2017). These observations support the hypothesis that leather source and degradation environment significantly shape microbial assemblages, even under standardised culture conditions.

At the taxonomic level, *Bacillus* was the dominant genus across most samples, consistent with its known role in collagen degradation, biofilm formation, and metal tolerance (He et al. 2018; Alotaibi et al. 2021; Wróbel et al. 2023). *Pseudomonas*, *Acinetobacter*, and *Ornithinibacillus* were prominent in several samples. The presence of *Lysinibacillus* and *Microbacterium*, identified metagenomically and through strain isolation, is particularly relevant due to their documented ability to reduce or tolerate chromium (VI) and participate in bioremediation (Sarkar et al. 2016; Ashraf et al. 2018; Abo Elazm et al. 2020; Ouertani et al. 2020; Mishra et al. 2021). In particular, the presence of *Microbacterium* supports its environmental relevance, as this genus has previously been linked to keratin degradation and chromate detoxification (Lun et al. 2016). The co‐detection of *Acinetobacter*, *Pseudomonas*, and *Ornithinibacillus* further aligns with the literature identifying these genera in heavy metal‐ or tannery‐impacted environments (Zakaria et al. 2007; Li et al. 2020; Sevak et al. 2023).

Cultivable strains recovered from samples ine12, ine14 and ine15 confirmed the presence of genera of biotechnological interest such as *Microbacterium*, *Brevibacterium*, and *Lysinibacillus*. Several genera, notably *Microbacterium* and *Bacillus*, have been reported to facilitate the biocementation and enzymatic degradation of leather and other recalcitrant protein‐based materials (Khambhaty 2020; Moonnee et al. 2021). Their high similarity to known strains from chromium‐contaminated environments or tannery effluents suggests a pre‐adaptation to harsh leather‐associated conditions. These isolates are promising candidates for future applications in controlled degradation and bioreactor settings.

Gravimetric analysis showed that approximately half of the samples achieved a measurable mass loss of 1% to 3% over 8 weeks. These moderate values are consistent with findings from standardised ISO 20136 tests and previous microbial degradation assays of wet blue leather (ISO 20136; Bonilla‐Espadas et al. 2024). Samples with the highest degradation often harboured abundant *Bacillus* or co‐occurring *Microbacterium*, *Ornithinibacillus*, and *Acinetobacter*, supporting the hypothesis that these genera play key roles in collagen degradation. Conversely, the samples such as ine4, ine11, ine12, ine13 and ine14 exhibited apparent mass gains. These anomalies, initially thought to result from EPS or biomass accumulation, persisted despite using a washing protocol involving SDS, ethanol, and sterile water.

Although the washing procedure was intended to remove all surface biofilms, the variability in leather porosity and microbial adhesion strength likely contributed to incomplete biomass removal. In samples with low or no measurable degradation, microbial communities may still metabolise accessible protein fractions without sufficient matrix breakdown to cause a significant mass loss. These findings reflect the challenge of interpreting early‐stage biodegradation in complex substrates and reinforce the need to combine gravimetric results with the structural and molecular indicators of microbial activity.

SEM analysis confirmed microbial colonisation and collagen matrix deterioration in several inoculated samples. Ine14 and ine15 displayed well‐developed, EPS‐rich biofilms and disruptions in fibrillar structure consistent with enzymatic degradation (Bozec and Odlyha 2011; Vyskočilová et al. 2022). Amorphous or gelatinised zones were observed at high magnification, particularly in ine15, suggesting advanced microbial digestion of leather fibres. In contrast, sample ine12 showed sparse bacterial clusters and no evidence of EPS formation, consistent with the minimal degradation and early colonisation stages.

The morphological simplification of the microbial populations observed under SEM, with reduced diversity in cell forms, may reflect consortia optimization under selective pressure for leather degradation. EPS‐producing taxa are especially relevant to these observations, as EPS enhance microbial adhesion, protect against metal toxicity, and support extracellular enzymatic activity (Cappello et al. 2011; Vasconcellos et al. 2011; Maksimova 2014; Staninska‐Pięta et al. 2020).

The control sample analysed by SEM retained its collagen ultrastructure and showed no microbial presence, confirming that the introduced environmental consortia initiated degradation. These findings reinforce the importance of microbial inoculation for initiating biodegradation and demonstrate that even minimal medium conditions can support early‐stage breakdown if suitable microbial communities are present. Altogether, this study showed that naturally derived microbial consortia can initiate the degradation of chromium‐tanned leather under minimal conditions. Although achieving complete degradation was not the aim of this study, the observed microbial activity and structural impact indicate the onset of a biodegradation process. The correlation between moderate mass loss and biofilm structures, particularly in ine14 and ine15, underscores the importance of microbial adhesion and enzymatic action in early‐stage degradation. However, the relationship between SEM observations and gravimetric mass loss is not always consistent. For example, sample ine12 showed sparse bacterial adhesion and no EPS structures despite its classification as a high‐potential inoculant, whereas ine15 displayed dense biofilm formation and matrix disruption. These discrepancies suggest that visible microbial colonisation alone does not fully account for degradative capacity and that other variables, such as enzymatic profiles or microbial metabolic state, may influence degradation dynamics.

## 5 Conclusions

This study demonstrates that naturally derived microbial consortia, selected from decomposing leather materials, can initiate the biodegradation of chromium‐tanned leather under nutrient‐limited conditions. While complete degradation was not the objective, evidence of microbial activity—including moderate mass loss, taxonomic profiles of degradation‐relevant genera (*Bacillus*, *Microbacterium*, *Ornithinibacillus*), and visible matrix disruption—supports the feasibility of biologically mediated leather breakdown.

The metagenomic analysis revealed diverse microbial communities enriched in taxa known for collagen degradation and metal tolerance. Complementary SEM analysis confirmed surface‐level microbial colonisation and biofilm formation, particularly in samples with greater structural deterioration. Gravimetric measurements further highlighted the potential for selected consortia to initiate material degradation despite the stabilising effects of chromium tanning.

Moreover, the successful isolation of cultivable strains with known biotechnological relevance lays the groundwork for targeted degradation trials and downstream applications in bioaugmentation or reactor‐based systems. This work provides foundational insight into the ecological and functional roles of complex microbial communities in the early stages of solid leather waste biodegradation. It supports further development of biologically based, sustainable waste management strategies for the leather industry.

## Author Contributions

**Marcelo Bertazzo** and **María‐José Bonete:** conceptualisation. **Manuela Bonilla‐Espadas** and **Irene Lifante‐Martínez:** data curation. **Manuela Bonilla‐Espadas:** formal analysis. **Elena Orgilés‐Calpena** and **María‐José Bonete:** funding acquisition. **Manuela Bonilla‐Espadas:** investigation. **Manuela Bonilla‐Espadas** and **Irene Lifante‐Martínez:** methodology. **Elena Orgilés‐Calpena, Francisca Arán‐Aís,** and **María‐José Bonete:** project administration. **Mónica Camacho:** resources. **Elena Orgilés‐Calpena, Francisca Arán‐Aís, Marcelo Bertazzo,** and **María‐José Bonete:** supervision. **Manuela Bonilla‐Espadas:** validation. **Manuela Bonilla‐Espadas:** writing – original draft. **Mónica Camacho, Marcelo Bertazzo,** and **María‐José Bonete:** writing – review and editing. All authors have read and agreed to the published version of the manuscript.

## Conflicts of Interest

The authors declare no conflicts of interest.

## Data Availability

The data that support the findings of this study are available from the corresponding author upon reasonable request.
